# Small-Aperture Monovision and the Pulfrich Experience: Absence of Neural Adaptation Effects

**DOI:** 10.1371/journal.pone.0075987

**Published:** 2013-10-14

**Authors:** Sotiris Plainis, Dionysia Petratou, Trisevgeni Giannakopoulou, Hema Radhakrishnan, Ioannis G. Pallikaris, W. Neil Charman

**Affiliations:** 1 Institute of Vision & Optics (IVO), University of Crete, Heraklion, Greece; 2 Faculty of Life Sciences, The University of Manchester, Manchester, United Kingdom; Massachusetts Eye & Ear Infirmary, Harvard Medical School, United States of America

## Abstract

**Purpose:**

To explore whether adaptation reduces the interocular visual latency differences and the induced Pulfrich effect caused by the anisocoria implicit in small-aperture monovision.

**Methods:**

Anisocoric vision was simulated in two adults by wearing in the non-dominant eye for 7 successive days, while awake, an opaque soft contact lens (CL) with a small, central, circular aperture. This was repeated with aperture diameters of 1.5 and 2.5 mm. Each day, monocular and binocular pattern-reversal Visual Evoked Potentials (VEP) were recorded. Additionally, the Pulfrich effect was measured: the task of the subject was to state whether a a 2-deg spot appeared in front or behind the plane of a central cross when moved left-to-right or right-to-left on a display screen. The retinal illuminance of the dominant eye was varied using neutral density (ND) filters to establish the ND value which eliminated the Pulfrich effect for each lens. All experiments were performed at luminance levels of 5 and 30 cd/m^2^.

**Results:**

Interocular differences in monocular VEP latency (at 30 cd/m^2^) rose to about 12–15 ms and 20–25 ms when the CL aperture was 2.5 and 1.5 mm, respectively. The effect was more pronounced at 5 cd/m^2^ (i.e. with larger natural pupils). A strong Pulfrich effect was observed under all conditions, with the effect being less striking for the 2.5 mm aperture. No neural adaptation appeared to occur: neither the interocular differences in VEP latency nor the ND value required to null the Pulfrich effect reduced over each 7-day period of anisocoric vision.

**Conclusions:**

Small-aperture monovision produced marked interocular differences in visual latency and a Pulfrich experience. These were not reduced by adaptation, perhaps because the natural pupil diameter of the dominant eye was continually changing throughout the day due to varying illumination and other factors, making adaptation difficult.

## Introduction

One way of improving the near vision of an emmetropic presbyopic eye is to increase its depth-of-focus by introducing a small artificial pupil.[1] This has led to increased interest in the use of such an approach in clinical work, the small aperture usually being confined to one eye, since the small pupil causes some restriction in visual field.[2], [3] However, although monocular use of small-aperture optics in the form of a corneal inlay or contact lens (CL), i.e. small-aperture monovision, may improve the intermediate and near binocular acuity of presbyopes,[4]–[13] it is obvious that less light contributes to the retinal image in the eye with the small, fixed pupil, so that the wearer may perform binocular visual tasks with more difficulty under mesopic or scotopic conditions, when there is large interocular difference in pupil sizes.

More interestingly, the interocular differences in retinal illuminance result in interocular differences in the latency of the Visual Evoked Potentials (VEP),[14] and the induced Pulfrich effect.[15] Such effects may potentially cause distortions in the perception of position and relative speed in situations where the visual environment is changing rapidly, such as driving or moving through a congested area[16]–[20] and hence may result in hazard. The important question therefore arises as to whether the individual can adapt to this aspect of the artificial anisocoria, thereby reducing such hazards.

The continued existence of the spontaneous Pulfrich effect in clinical patients argues that complete adaptation does not occur in such cases.[17] When the Pulfrich effect is induced by placing a neutral density filter over one eye, partial adaptation does occur: the effect may reduce by a factor of about 3 over a one-week period.[21] Similar adaptation has been reported during prolonged monocular wear of the spectrally-selective X-Chrom contact lens by subjects with normal or anomalous colour vision.[22] However, in such situations the ratio between the retinal illuminances in the two eyes remains constant, since although the two natural pupil diameters may change with the ambient illumination, they remain equal. In contrast, when the difference between the two retinal illuminances is created by the use of a monocular artificial pupil their relationship is changing continually as the ambient illumination and diameter of the natural pupil change, which may make adaptation more difficult. The present study explores whether in this situation adaptation effects can reduce both the differences in VEP latency and the induced Pulfrich effect.

## Methods

### Small-aperture lenses

The effects of small-aperture monovision were simulated by using two afocal, hand-painted opaque soft contact lenses (74% water content), supplied by Cantor & Nissel Ltd, Brackley, UK) with a central circular aperture diameter of 1.5 and 2.5 mm. The outer diameter of the opaque region was 8.0 mm. The lenses were inserted in the non-dominant eye.

### Experimental Procedure

Two normal adult subjects [aged 42 (SP) and 32 (TG)], occasional contact lens-wearers, with visual acuity higher than 1.0 in decimal notation, best-corrected for distance, participated in the study. Subjects wore each of the contact lenses during all waking hours (about 16–18 hours a day) for seven successive days. They were asked to refrain from driving during the period of the study and provided a written consent prior to their participation. The study was conducted in adherence to the tenets of the Declaration of Helsinki and followed a protocol approved by the University of Crete Research Board.

Monocular and binocular VEPs and Pulfrich recordings were measured at a specific time (12 to 2 pm) each day: Day 0 (15 minutes following lens insertion), Day 1, Day 2, Day 3, Day 4, Day 6 (only for subject TG) and Day 7. Baseline recordings were also performed with natural pupils one day before (Day -1) and after (Day 8) the CL wearing period. All measurements were performed with best-corrected vision for distance at luminance levels of 5 and 30 cd/m^2^. No mydriatics or cycloplegics were used.

The monocular VEP measurements were made on the lens-wearing, non-dominant eye with the dominant eye being covered with an eye patch, and on the dominant eye with the non-dominant eye being patched. Eye dominance was determined by looking through a central hole in an A4 card, held by the participant in both hands away from the body.

Average pupil diameters during the recordings in each condition were measured with head-mounted infrared cameras (EyeLink II, SR Research Ltd., Canada), which provided a magnification of approximately 4.5×.

### VEP recordings

Recordings of visual evoked potentials (VEPs) took place in a sound-attenuated room with the lights off. VEPs were elicited using reversing 10 arcmin achromatic checks (nominal dominant spatial frequency 3 c/deg) with 100% contrast, at a rate of 4 reversals per second (2 Hz) with a square-wave temporal modulation. The stimulus was displayed on a Sony GDM F-520 CRT monitor by means of a VSG 2/5 stimulus generator card (Cambridge Research Systems Ltd, UK) and a software tool which allowed space-averaged screen luminance to be controlled to either 30 or 5 cd/m^2^ (Visual Psychophysics Engine, Cambridge Research Systems Ltd, UK). At the 1.0 m testing distance, the stimulus subtended a circular field of 15 degrees diameter. Fixation was achieved using a centrally-placed cross.

VEPs were recorded using silver-silver chloride electrodes. An active electrode was positioned 10% of the distance between the inion and the nasion over the vertex and referenced to an electrode placed at Fz with a ground electrode placed on the forehead. The active and reference electrodes were applied to the head with electrode paste after the area had been thoroughly cleaned. Trigger synchronisation was achieved using a CED 1401 “micro” (Cambridge Electronic Design, UK). The waveforms were amplified (gain = 10 K) using the CED 1902 (Cambridge Electronic Design, UK). Amplifier bandwidth was set at 0.5–30 Hz (together with a 50 Hz notch filter) and signals were sampled at a rate of 1024 Hz with an analysis time of 0.970 s. Data acquisition and averaging were controlled using the Signal software (vs. 3.1, CED, UK). Each VEP trace was the average of 64 epochs of 1 sec duration each, as suggested by the International Society of Clinical Electrophysiology of Vision (ISCEV).[23] Computerized artifact rejection was performed before signal-averaging, according to standard ISCEV guidelines, in order to discard epochs in which deviations in eye position, blinks, or amplifier blocking occurred.

P100 peak amplitude and latency (time) were derived from the average waveform. This required manual definition of the lowest negative peak (N75) prior to the P100 peak. Amplitude was scored as the voltage difference between these two points and latency as the time difference between the P100 peak and stimulus onset.

### Pulfrich effect

The Pulfrich stimulus was a circular red spot of angular diameter 2.0 degrees moving sinusoidally in a horizontal direction on a display screen, with a period of 3 seconds and an amplitude of 11.25 degrees. The luminances of the spot and its achromatic background were either 5 and 30 cd/m^2^, respectively, or 0.83 and 5 cd/m^2^. The display, which also incorporated a large black fixation cross (angular dimensions: 1.5 deg), was placed at a distance of 0.4 m from the seated subject, whose head position was stabilized with a headrest. The resultant full angular subtense of the display was 45.3×37.3 degrees. The two-alternative, forced-choice task of the subject was to state whether, during binocular observation, the spot appeared in front or behind the plane of the fixation cross when the spot moved left-to-right or right-to-left.

The retinal illuminance of the dominant eye, having a natural pupil, was varied by using 13 combinations of neutral density (ND) filters, ranging from 0.13 to 1.48 ND in approximately 0.11 ND intervals, together with the “no filter” condition. With the non-dominant eye wearing each of the 2 contact lenses, filters were presented in random order in front of the dominant eye. A total of 15 tests were carried out with each filter, to allow a psychometric function to be plotted. This was fitted with a cumulative Weibull distribution function of the form P = 1−exp(−10?b(x−t), where P is the response probability, x is the ND value and b and t are the parameters that define the threshold (in ND) and the slope of the Weibull function, respectively. The ND corresponding to P = 0.50 was taken as the value required to counteract the artificial anisocoria.

## Results

Subjects reported a difficulty in accomplishing near vision tasks under mesopic conditions.

### VEP experiments

**Figure 1:**
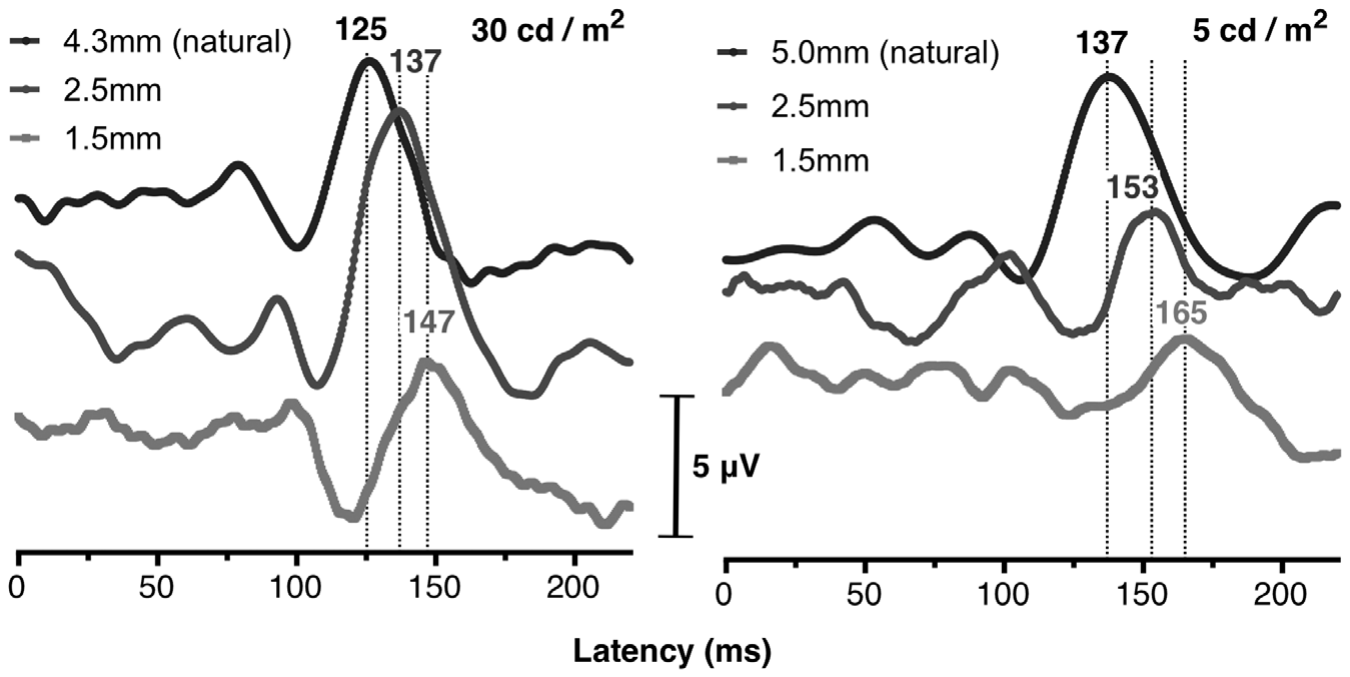
Effect of CL aperture luminance levels on a characteristic VEP waveform. Grand-averaged (64 epochs) monocular VEP waveforms from one subject at high (30 cd/m^2^, left) and low (5 cd/m^2^, right) photopic levels for a natural pupil (black line) and with a contact lens of 2.5 mm (dark grey line) and 1.5 mm (light grey line) aperture. P100 latency is indicated in ms.

Figure 1 depicts characteristic monocular VEP waveforms of the non-dominant eye with natural pupil and with the contact lenses of 2.5 and 1.5 mm aperture diameter at two luminance levels. It is evident that the P100 component of the Visual Evoked Potential (VEP) yields a longer latency for the smaller pupil in both conditions, in agreement with previous studies.[14], [24], [25] VEP P100 peak latency is also delayed for the lower (5 cd/m^2^) screen luminance in comparison to the 30 cd/m^2^ level. Note that the amplitude of the P100 component is also affected by pupil size and luminance but this is less consistent, in agreement with the literature suggesting that P100 peak amplitude shows higher variation between and within subjects compared to the P100 peak latency.

**Figure 2:**
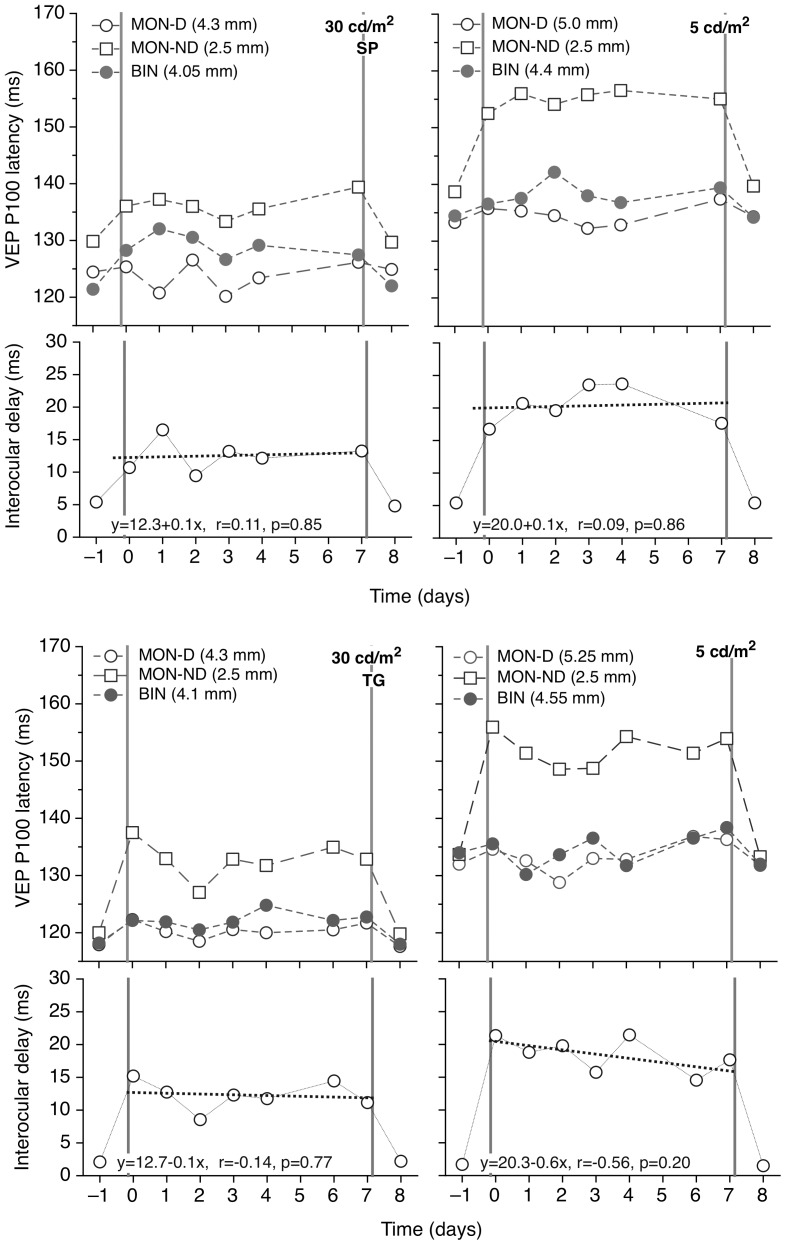
Plots of VEP latency as a function of time for the 2.5. (upper) Monocular (open circles, dominant eye; open squares, non-dominant eye) and binocular (filled circles) mean latency of the VEP P100 component as a function of time at high (left) and low (right) photopic levels for subjects SP (fig 2a) and for TG (fig 2b). Each point is the average of two recordings. On days -1 and 8 the subject had unobstructed natural pupils. On days 0–7 inclusive the non-dominant (left) eye was wearing a contact lens with an aperture of 2.5 mm in diameter. The dominant eye had its full, unobstructed natural pupil. The legend shows the average pupil size at each condition. (lower) Plot of the interocular latency difference as a function of time. The dotted bold line forms a linear regression for Days 0 to 7.

**Figure 3:**
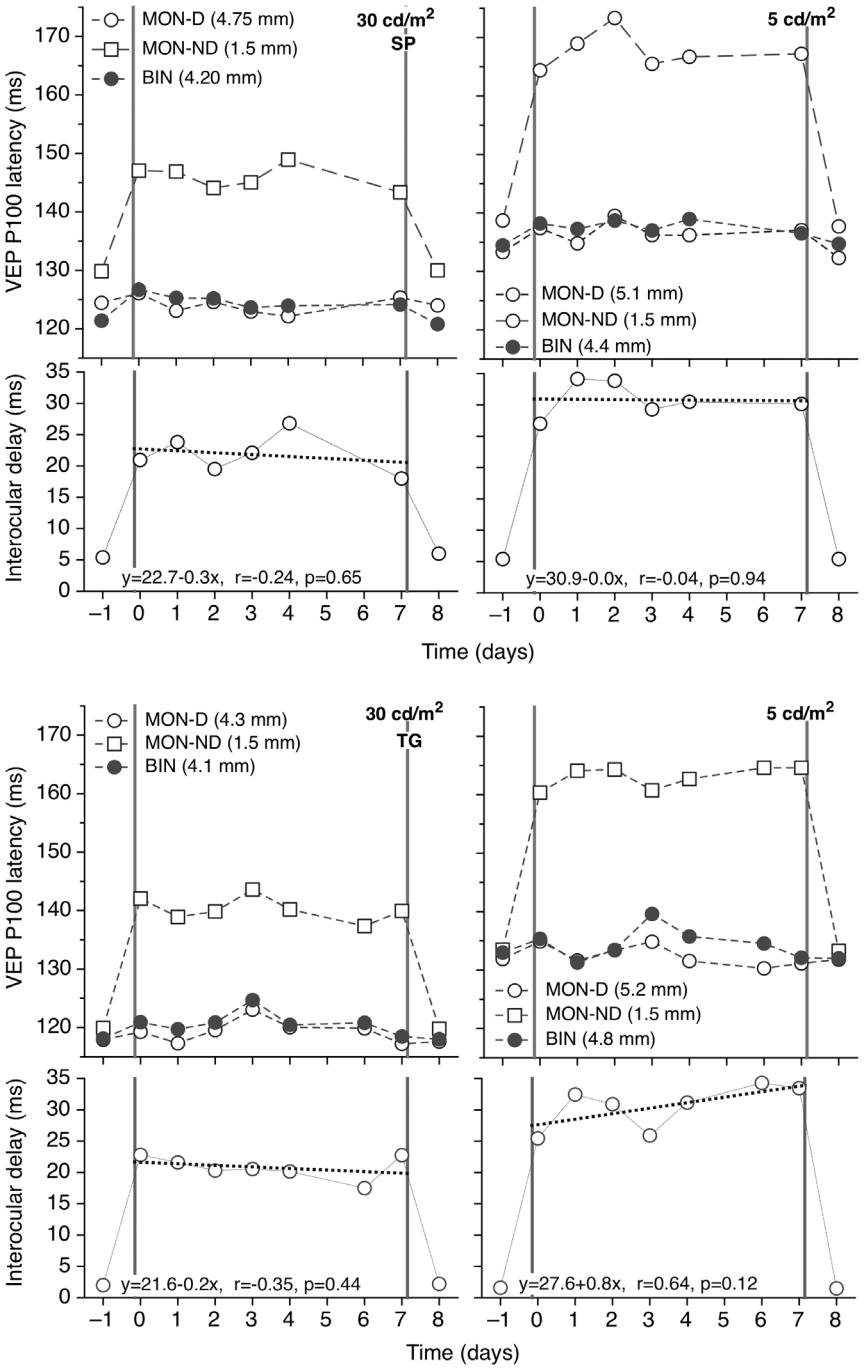
Plots of VEP latency as a function of time for the 1.5. (upper) Plots of monocular (open circles, dominant eye; open squares, non-dominant eye) and binocular (filled circles) mean latency of the VEP P100 component as a function of time at high (left) and low (right) photopic levels for subjects SP (fig 3a) and TG (fig 3b). Each point is the average of two recordings. On days -1 and 8 the subject had unobstructed natural pupils. On days 0–7 inclusive the non-dominant (left) eye was wearing a contact lens with an aperture of 1.5 mm in diameter. The dominant eye had its full, unobstructed natural pupil. The legend shows the average pupil size at each condition. (lower) Plot of the interocular latency difference as a function of time. The dotted bold line forms a linear regression for Days 0 to 7.

The upper parts of figures 2 and 3 plot the P100 latency of the Visual Evoked Potential (VEP) between day -1 and day 8 for the two subjects, when tested under various pupil and luminance conditions. Insertion of either of the reduced aperture CLs markedly increased the monocular VEP latency. The effect was more pronounced at the lower screen luminance and with the smaller artificial pupil. The effect of monocular introduction of the artificial pupils on the binocular latencies was minor and not statistically significant.

The lower parts of the figures show the interocular differences in monocular VEP latency. At 30 cd/m^2^, when the pupil of the non-dominant eye was either 2.5 mm or 1.5 mm, these differences rose on average to about 12–15 ms and 20–25 ms, respectively. At 5 cd/m^2^, when the pupil of the non-dominant eye was either 2.5 or 1.5 mm, inter-ocular differences in VEP latency rose to about 20 ms and 30 ms respectively.

Figures 2 and 3 show no indication of any reduction in the interocular differences in VEP latency during the seven days of small-aperture CL wear for both subjects. In no case does the gradient of the regression line fit to the plot of interocular latency delay vs. period of adaptation differ significantly from zero. Following CL removal, all VEP latencies return to pre-adaptation values.

### Pulfrich experiments

**Figure 4:**
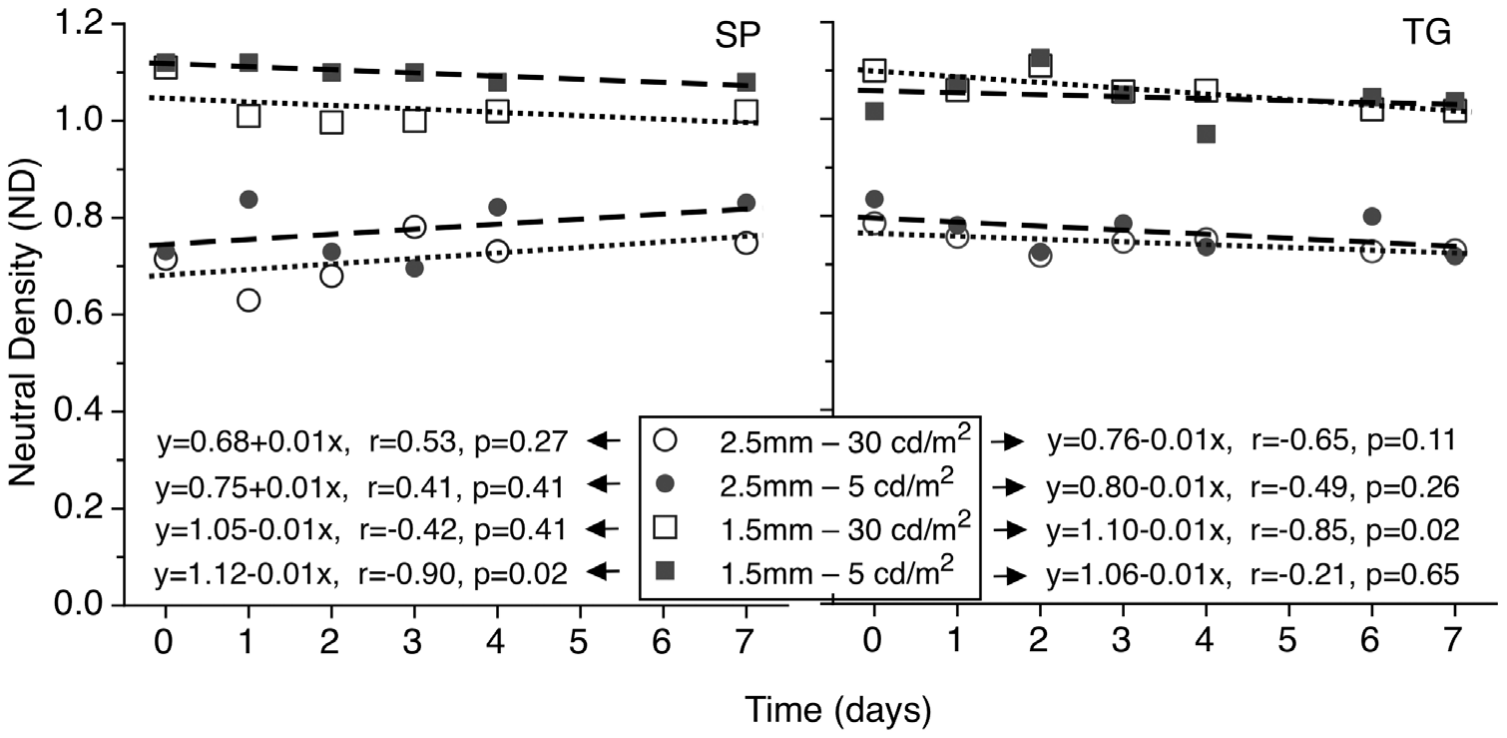
Values of ND filter required to null the Pulfrich effect as a function of time. The non-dominant eye was wearing a 2.5 mm (circles) or a 1.5 mm lens (squares). Data for subject SP (left) and TG (right) at high (open symbols) and low (filled symbols) photopic levels are presented. The average values of ND filter required to null the Pulfrich effect, when placed in front of the dominant eye, for all conditions are also shown. The dotted / dashed lines form a linear regressions for Days 0 to 7.

A strong Pulfrich effect was observed with both lenses under all conditions. Fig.4 shows the null values of ND over the 7-day period of anisocoric vision. The ND values required to null the effect were higher for the smaller aperture case (1.5 mm) than those for the larger aperture (2.5 mm). NDs for 1.5 mm pupil cases varied over the trial period between 0.64 to 0.75 and 0.72 and 0.79 for the two subjects, in comparison to the corresponding 2.5 mm NDs of between 0.98 and 1.10 and 1.08 and 1.12. The slopes of the linear regression fits to the ND vs. time data did not differ significantly from zero (i.e. the ND value required to null the Pulfrich effect did not reduce over the period of trial), indicating the absence of any adaptation effects. Following CL removal at the end of their 7-day wearing period, the Pulfrich effect disappeared for both subjects.

For both the 1.5 and 2.5 mm apertures, no significant differences were found between the Pulfrich effects experienced at the two screen luminance levels.

## Discussion

The anisocoria induced by small-aperture CLs produced marked interocular differences in visual latency and a Pulfrich experience. These differences were not reduced by any short-term adaptation, perhaps because the natural pupil diameter of the dominant eye was continually changing throughout the day due to changing illumination and other factors (e.g. accommodative vergence[26] and mental or emotional state[27]), making adaptation difficult.

**Figure 5:**
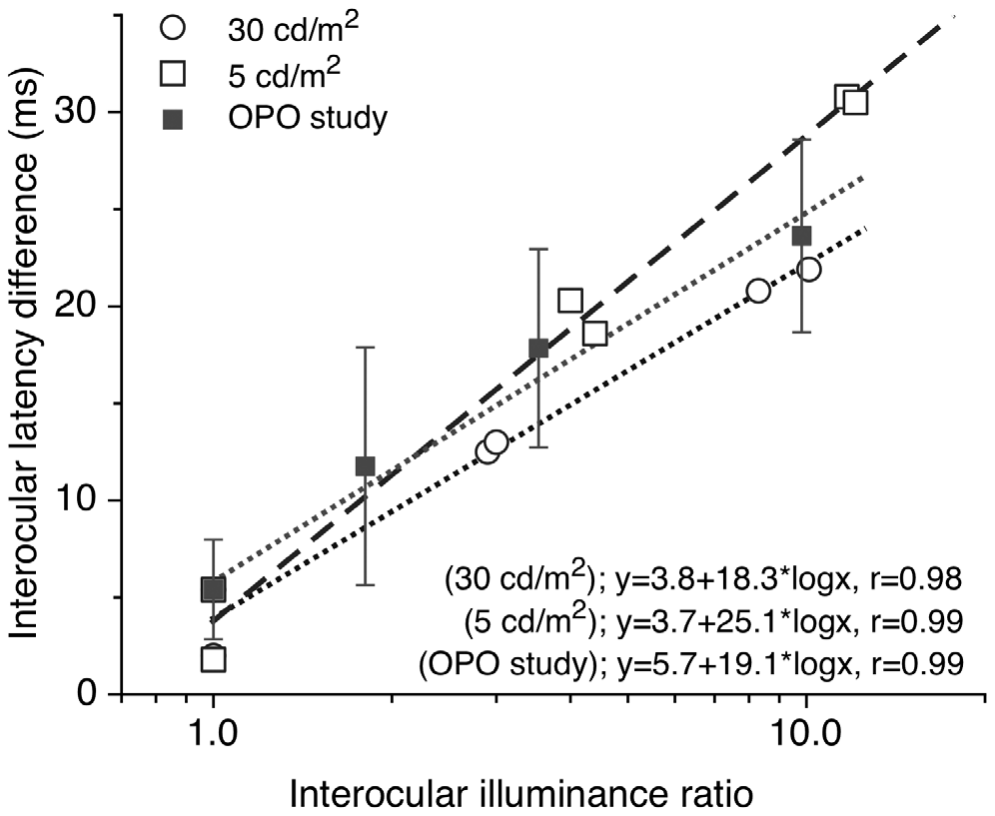
Interocular differences in VEP latency as a function of the interocular ratio of retinal illuminance. VEP latency is averaged for recordings between Day 0 and Day 7 and is plotted for two photopic luminance levels (30 vs. 5 cd/m^2^). The results of an earlier study[14] using 7 subjects are shown for comparison. The bars indicate ±1 SD.

The observed increased interocular delay at low luminance levels (Figures 2 and 3) can be explained by the higher ratio of the greater to the lesser of the retinal illuminances in the two eyes associated with the various pupil diameters involved (see Table 1). This interocular illuminance ratio, calculated from the relative pupil areas, is about 2.9 and 3.0 when using the 2.5 mm aperture lens at 30 cd/m^2^ for subjects SP and TG, respectively. It increases to 4.0 and 4.4 at 5 cd/m^2^. For the 1.5 mm lens the interocular ratio is higher, leading to longer interocular delays. These changes are summarized in Figure 5, which also includes earlier data based on the average of responses for 7 subjects at 30 cd/m^2^ and additional artificial pupil diameter.[14] The interocular latency difference increased linearly with the logarithm of the interocular illuminance ratio, as also suggested by previous studies.[25], [28] The slope of these functions is higher for the low (slope 25.1) compared to the high (slope 19.1, 18.3[14] and 16[25]) photopic conditions, possibly due to post-retinal neurophysiological processing. Note that when the interocular illuminance ratio is unity (no anisocoria) the interocular latency difference is not zero, i.e. dominant eye produces slightly faster responses.

**Table 1:** Νatural pupil diameter (in mm) during binocular / monocular VEP recordings and for two luminance levels.

Viewing condition	Subject SP		Subject TG	
	Binocular	Monocular	Binocular	Monocular
2.5 mm – 30 cd/m^2^	4.05	4.29 (2.9)	4.09	4.32 (3.0)
2.5 mm – 5 cd/m^2^	4.40	5.00 (4.0)	4.55	5.23 (4.4)
1.5 mm – 30 cd/m^2^	4.17	4.76 (10.1)	4.09	4.32 (8.3)
1.5 mm – 5 cd/m^2^	4.40	5.12 (11.7)	4.77	5.23 (12.1)

The non-dominant eye was wearing a contact lens with an aperture of 2.5 or 1.5 mm in diameter. The values in parentheses correspond to the interocular illuminance ratio, i.e. the ratio of the retinal illuminance in the dominant eye to that of the non-dominant eye, calculated from the relative pupil areas.

Another interesting observation is the delay in the P100 peak in all conditions (for both subjects) under binocular observation with anisocoric vision compared to monocular (dominant eye) observation. It is expected that a smaller pupil with binocular vision would have some effect, since P100 peak latency gets shorter with increasing pupil size (i.e. with increasing retinal illuminance),[14], [25] However, the delay is more evident with the 2.5 mm rather than the 1.5 mm aperture contact lens and does not increase at low photopic levels, as the above hypothesis would imply, suggesting an inhibitory binocular interaction when the interocular difference is relatively low.[29] With higher degrees of binocular imbalance (higher interocular differences), which occur with the combination of the 1.5 mm aperture CL and the dilated natural pupil under low luminance conditions, the inhibition is less evident.

**Figure 6:**
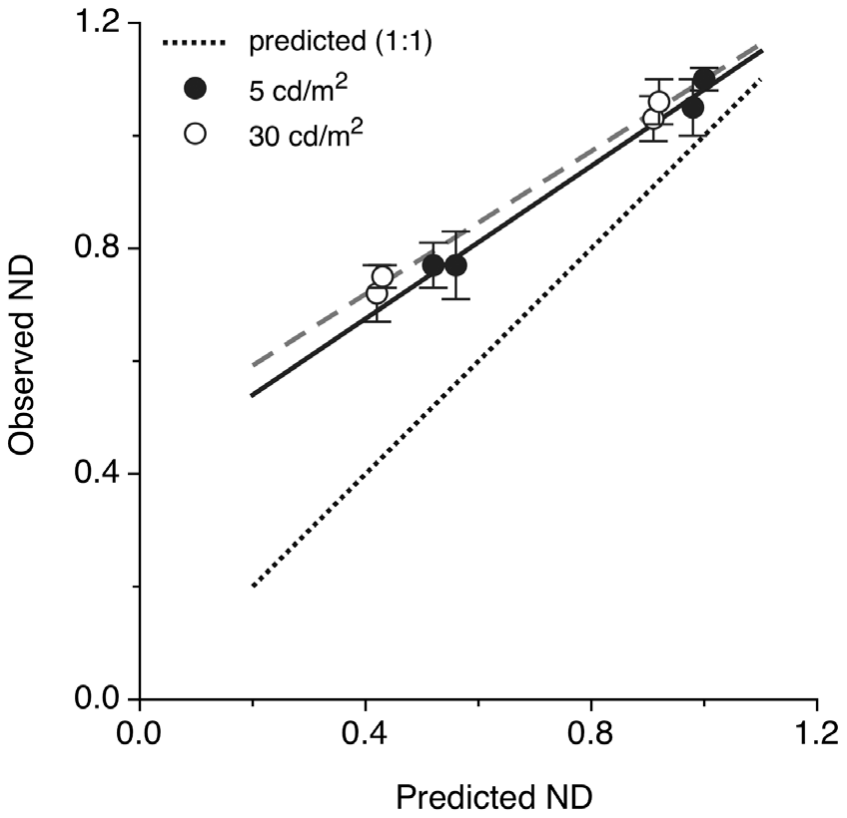
Observed vs. predicted values of ND filter required to null the Pulfrich effect. Average data are shown for the 2 observers and the 2 luminance conditions. Predicted ND values are based simply on relative pupil areas. The non-dominant eye was wearing a small-aperture CL of 1.5 or 2.5 mm in diameter. The dotted line represents exact agreement with predictions. The dashed lines form regression fits. The bars indicate ±1 SD during the 7 days of the trial.

In the Pulfrich measurements, a simple prediction of the nulling ND values, ND_null_, which might be expected can be made on the basis of the ratio of the retinal illuminances in the two eyes (i.e. ND_null_ = log_10_R, where R is the pupil area in the dominant eye divided by that in the non-dominant, CL-wearing eye). Average pupil diameter values recorded during the Pulfrich experiment, are shown in Table 2. Fig.6 shows that, in general, observed ND values are higher than the predicted ND values, an effect which is more pronounced with the larger artificial pupil. This discrepancy may be due to the nature of the Pulfrich experiment. Placing ND filters in front of the dominant eye (having a natural pupil), during the experiment reduces the total amount of light reaching the retina and causes a dilation in the natural pupil which increases with the ND value, although constant retinal illuminance is not maintained. This tends to increase the observed null ND value in comparison with that predicted on the basis of an average natural pupil diameter. Moreover, when the artificial pupil is larger, and hence has a diameter closer to that of the natural pupil, partial vignetting may occur at smaller field angles, due to relative decentration of the clear aperture of the contact lens with respect to the artificial pupil. This is expected to create a requirement for increased filter density in front of the dominant eye. Such effects do not alter the conclusion that no adaptation was observed over the 7-day CL wearing period.

**Table 2:** Νatural pupil diameter (in mm) of the dominant eye during Pulfrich recordings.

Viewing condition	Subject SP	Subject TG
2.5 mm – 30 cd/m^2^	4.05 (2.2)	4.09 (2.1)
2.5 mm – 5 cd/m^2^	4.76 (3.6)	4.55 (3.3)
1.5 mm – 30 cd/m^2^	4.29 (6.1)	4.32 (6.3)
1.5 mm – 5 cd/m^2^	4.76 (10.1)	4.66 (9.7)

The non-dominant eye was wearing a contact lens with an aperture of 2.5 or 1.5 mm in diameter. The values in parentheses correspond to the interocular illuminance ratio, i.e. the ratio of the retinal illuminance in the dominant eye to that of the non-dominant eye, calculated from the relative pupil areas.

It is of interest that changing the stimulus luminance appeared to have less effect upon the Pulfrich data (Fig.4) than on the VEP delays (Figs 2 and 3). However, the neural pathways for the VEP and Pulfrich effect are different[30], [31] and others have found that interocular VEP delays are much longer than those deduced from the Pulfrich effect and that the two latencies may not always be simply correlated.[18]

Previous studies have also demonstrated that subjective reports are not an adequate basis for assessing the spatial distortions caused by the Pulfrich effect. More objective measures show that, while there is distortion of the apparent track of a moving target, it is not as great as the subjective reports indicate.

Moreover, sustained or repeated exposure to the same conditions may be particularly deceptive. In some situations in which there may be a disparity of light intensity inputs in the two eyes, caution is also indicated. For example, if one is driving a vehicle south and the sun is setting in the west, the bridge of the nose could occlude light from the setting sun to the left eye and not to the right eye. The resulting disparity of intensities input to the two eyes could lead to unpredictable errors in depth judgement.

Finally, small artificial pupils are expected to result in peripheral visual field loss.[2], [3] Since in small-aperture monovision the artificial pupil is confined to one eye, if a simple circular pupil is used some depression in binocular peripheral sensitivity is expected, rather than a full scotoma, except for the temporal monocular crescent on the side of the eye with the artificial pupil where the scotoma may be more complete. The position is more complex with monovision using current designs of small-aperture corneal inlay^5–13^, in which the opaque area of the artificial pupil is annular, with inner and outer radii 0.8 and 1.9 mm respectively. Although no studies have dealt with this issue, the impact of the inlay on the peripheral field is expected to depend not only on the geometry of the inlay but also on the natural pupil diameter, with the effect on the field being more pronounced the smaller the natural pupil. Clinical studies suggest that the central field is essentially normal^9^.

## Conclusions

The interocular differences in retinal illuminance associated with reduced aperture monovision cause differences in visual latency and a Pulfrich effect. These do not appear to be reduced by adaptation. These effects may lead to distortions in the perception of relative movement and, in some cases, to possible hazard in practical situations such as driving. It is to be expected that broadly similar effects would be found if the reduced aperture was placed in a corneal inlay or an intraocular lens.
